# Atypical properties of a conventional calcium channel β subunit from the platyhelminth *Schistosoma mansoni*

**DOI:** 10.1186/1472-6793-8-6

**Published:** 2008-03-26

**Authors:** Vicenta Salvador-Recatalà, Toni Schneider, Robert M Greenberg

**Affiliations:** 1 Marine Biological Laboratory 7 MBL Street Woods, Hole, MA 02543, USA; 2 Institute for Neurophysiology & Center for Molecular Medicine Cologne (CMMC) Robert-Koch-Str. 39 D-50931 Köln, Germany; 3 Department of Pathobiology, School of Veterinary Medicine, University of Pennsylvania, 3800 Spruce Street, Philadelphia, PA 19104, USA

## Abstract

**Background:**

The function of voltage-gated calcium (Ca_v_) channels greatly depends on coupling to cytoplasmic accessory β subunits, which not only promote surface expression, but also modulate gating and kinetic properties of the α_1 _subunit. Schistosomes, parasitic platyhelminths that cause schistosomiasis, express two β subunit subtypes: a structurally conventional β subunit and a variant β subunit with unusual functional properties. We have previously characterized the functional properties of the variant Ca_v_β subunit. Here, we focus on the modulatory phenotype of the conventional Ca_v_β subunit (SmCa_v_β) using the human Ca_v_2.3 channel as the substrate for SmCa_v_β and the whole-cell patch-clamp technique.

**Results:**

The conventional *Schistosoma mansoni *Ca_v_β subunit markedly increases Ca_v_2.3 currents, slows macroscopic inactivation and shifts steady state inactivation in the hyperpolarizing direction. However, currents produced by Ca_v_2.3 in the presence of SmCa_v_β run-down to approximately 75% of their initial amplitudes within two minutes of establishing the whole-cell configuration. This suppressive effect was independent of Ca^2+^, but dependent on intracellular Mg^2+^-ATP. Additional experiments revealed that SmCa_v_β lends the Ca_v_2.3/SmCa_v_β complex sensitivity to Na^+ ^ions. A mutant version of the Ca_v_β subunit lacking the first forty-six amino acids, including a string of twenty-two acidic residues, no longer conferred sensitivity to intracellular Mg^2+^-ATP and Na^+ ^ions, while continuing to show wild type modulation of current amplitude and inactivation of Ca_v_2.3.

**Conclusion:**

The data presented in this article provide insights into novel mechanisms employed by platyhelminth Ca_v_β subunits to modulate voltage-gated Ca^2+ ^currents that indicate interactions between the Ca^2+ ^channel complex and chelated forms of ATP as well as Na^+ ^ions. These results have potentially important implications for understanding previously unknown mechanisms by which platyhelminths and perhaps other organisms modulate Ca^2+ ^currents in excitable cells.

## Background

Voltage-gated calcium (Ca_v_) channels couple membrane depolarisation to the entry of Ca^2+ ^that, in turn, is fundamental in a variety of cellular events such as contraction [1,2], changes in gene expression [3] and neurotransmitter release [4,5]. Ca_v _channels belong to the super-family of voltage-gated ion channels that also include sodium channels and potassium channels [6], and can be broadly classified into high-voltage activated (HVA) and low-voltage activated (LVA) classes. HVA Ca_v _channels are heteromeric protein complexes composed of a pore-forming α_1 _subunit and auxiliary β and α_2_δ subunits [7]. In addition to promoting surface expression of the Ca_v_α_1 _subunit, Ca_v_β subunits modulate the kinetics of activation and inactivation, gating [8-10] and the rate of recovery from inactivation [11,12].

Schistosomes are parasitic trematode flatworms that cause schistosomiasis, a tropical disease affecting approximately 200 million people worldwide. With the ultimate goal of understanding the molecular basis for neuromuscular transmission in these parasitic flatworms, we have previously cloned three transcripts from *Schistosoma mansoni *that code for one L-type-like and two non L-type high voltage-activated Ca_v _channel α_1 _subunits [13]. Heterologous expression of these α_1 _subunits in *Xenopus *oocytes and mammalian cell lines has proved problematic, perhaps because of the relatively high A-T content of these coding regions, or the lack of a specific chaperone in these systems. Additionally, we have identified two Ca_v _channel β subunits from schistosomes and other platyhelminths: a conventional β subunit (SmCa_v_β), and a variant β subunit (SmCa_v_β_var_), which appears to be unique to platyhelminths and has unusual structural and functional features [14,15].

When heterologously expressed in *Xenopus *oocytes, the conventional schistosome Ca_v_β subunit significantly increases Ca_v_2.3 current amplitude, and shifts the steady state inactivation curve to more hyperpolarized potentials [16] (in these experiments, we use the robustly expressing human Ca_v_2.3 α_1 _subunit as a "reporter" to assess β subunit function). The actions of this β subunit are consistent with those of mammalian Ca_v_β subunits [10]. Here, we have reproduced and extended our previous data on modulation of Ca_v_2.3 currents by the schistosome SmCa_v_β subunit (SmCa_v_β) in a mammalian cell system, which may better approximate the cellular milieu in which these channels are found *in situ*. *Xenopus *oocytes are widely used in expression of ion channels and other proteins, in part because they are primed for high levels of protein translation. However, most other cells are not that strongly geared towards this role. Oocytes are much larger than adult, differentiated cells and contain high amounts of yolk granules. In addition, the mammalian cell line HEK does not express the endogenous Ca_v_β subunit that complicates analysis of heterologously expressed Ca_v _channels in *Xenopus *oocytes [17]. Finally, adult *S. mansoni *live in a mammalian host environment.

Interestingly, during these studies, we observed a rapid run-down of the currents produced by Ca_v_2.3 channels co-expressed with this schistosome Ca_v_β subunit. Decrease of Ca^2+ ^channel activity under whole-cell patch-clamp, a configuration of the patch-clamp technique that disrupts the contact between membrane and cytoplasm, is a well-known phenomenon [18-21]. However, very few studies have dealt with the structural and biochemical causes for run-down, and those studies focus primarily on L-type Ca^2+ ^channels. Notably, Kameyama and collaborators were able to relate run-down of L-type Ca^2+ ^channels to the Ca^2+^-binding protein calmodulin [22]. Here we investigate the mechanism of this β subunit-dependent rundown, examining the role of several forms of ATP and cations in run-down of the Ca_v_2.3/SmCa_v_β complex. Additionally, we use a truncated β subunit protein to provide clues regarding the molecular substrate within the SmCa_v_β subunit that mediates run-down of Ca_v_2.3/SmCa_v_β currents.

## Results

### SmCa_v_β modulates activation and inactivation of Ca_v_2.3 in a conventional manner

**Figure 1:**
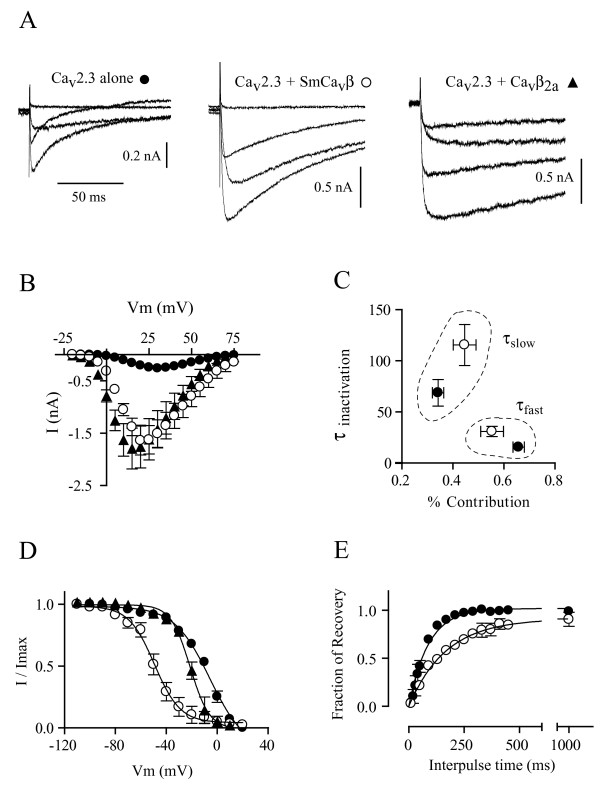
**SmCa_v_β modulates Ca_v_2.3 currents in HEK cells**. *A*. Representative Ca^2+ ^currents generated by voltage steps to -5, +15, +35 and +55 mV from a holding potential of -80 mV in HEK cells expressing Ca_v_2.3 alone and in combination with SmCa_v_β or Ca_v_β_2a_. *B*. Current-voltage relationships from HEK cells expressing Ca_v_2.3 alone (filled circles) and in combination with SmCa_v_β (open circles) or Ca_v_β_2a _(filled triangles). Peak currents generated by voltage steps from -20 mV to +75 mV in 5 mV steps from a holding potential of -80 mV were measured. Data points represent mean ± s.e.m. N = 5–15. *C*. Two-variable plot showing the relationships between inactivation time constants and percentages of contribution to total inactivation for Ca_v_2.3 alone (filled circles) and in combination with SmCa_v_β (open circles). Data points represent mean ± s.e.m. N = 7–9. Slow time constants for the kinetic of recovery are grouped (dotted lines). Fast time constants of recovery from inactivation kinetics are also grouped (dotted lines). *D*. Voltage-dependence of inactivation (steady-state inactivation) for Ca_v_2.3 alone (filled circles) and in combination with SmCa_v_β (open circles) or Ca_v_β_2a _(filled triangles). Lines represent fits to the Boltzmann function (see Materials and Methods); V_50 _and slope values are shown in Table 1. Data points represent mean ± s.e.m. N = 3–6. *E*. Recovery from inactivation from -100 mV for Ca_v_2.3 channels alone (filled circles) or in the presence of SmCa_v_β (open circles), using Ba^2+ ^as the charge carrier. Solid curves represent single exponential fits to the data. Values are means ± s.e.m. N = 3.

To assess the modulatory phenotype of Ca_v_β subunits, a HEK-293 cell line stably expressing the human Ca_v_2.3d subunit (GB Acc. # L27745) was used. Using Ca^2+ ^as the charge carrier, currents produced by Ca_v_2.3 alone peaked at +30 mV with an average amplitude of -261 ± 20 pA (n = 15). Co-expression of SmCa_v_β increased peak amplitudes to -1640 ± 276 pA (n = 5) and shifted the I–V peak leftwards, to +20 mV (Figure 1A,B).

**Table 1:** SmCa_v_β modulates inactivation of Ca_v_2.3 currents

	Ca_v_2.3 alone	Ca_v_2.3 + SmCa_v_β
*Steady state inactivation*		
V_50 _(mV)	-8 ± 4 (3)	-49 ± 3 (3)*
Slope (mV/e-fold)	12 ± 2 (3)	10 ± 1 (3)
*Macroscopic inactivation*		
τ_fast _(ms)	16 ± 3 (9)	31 ± 5 (7)*
% Contribution	66 ± 2%	55 ± 5%*
τ_slow _(ms)	69 ± 13 (9)	116 ± 20 (7)
% Contribution	34 ± 2%	45 ± 5%*
*Recovery from inactivation*		
τ_rec-80 mV _(ms)	178 ± 28 (3)	252 ± 17 (4)
τ_rec-100 mV _(ms)	71 ± 7 (3)	168 ± 16 (3)*
τ_rec-120 mV _(ms)	38 ± 1 (3)	109 ± 10 (3)*

Data represent mean ± s.e.m. Number of repeats is indicated in parentheses. Asterisks indicate statistically significant difference (Student t test, P < 0.05).

Using Ca^2+ ^as the charge carrier, the decaying phase of Ca_v_2.3 currents produced by Ca_v_2.3 channels alone or with SmCa_v_β subunits was well fitted by a double exponential function with fast (τ_fast_) and slow (τ_slow_) time constants of inactivation (Table 1). The double exponential fit to the decay of currents produced by Ca_v_2.3 in response to a pulse to +30 mV had a fast time constant of 16 ms that contributed 66% to total current decay, and a slow time constant of 69 ms that represented 34% of total current decay. The double exponential fit to the decay of currents produced by Ca_v_2.3 co-expressed with SmCa_v_β in response to a depolarizing pulse to +20 mV had a fast time constant of 31 ms, which contributed 55% to total current decay, and a slow time constant of 116 ms that contributed 45% to total inactivation. Fundamentally, SmCa_v_β slowed macroscopic inactivation of Ca_v_2.3 currents by increasing the time constants of both the fast and slow components while simultaneously decreasing the contribution of the fast component and increasing the contribution of the slow component to total inactivation (Figure 1C and Table 1). Co-expression of SmCa_v_β markedly shifted the midpoint of steady-state inactivation in the hyperpolarizing direction (Figure 1D). Midpoints of single order Boltzmann fits to steady-state inactivation curves of Ca_v_2.3 and Ca_v_2.3 + SmCa_v_β currents were, respectively, -8 mV and -49 mV (P < 0.05, Student t-test). The slope factors of these Boltzmann fits to steady-state inactivation were not significantly different: 12 mV/e-fold for Ca_v_2.3 and 10 mV/e-fold for Ca_v_2.3 + SmCa_v_β (Table 1).

SmCa_v_β significantly slowed the rate of recovery from inactivation. The fractional recoveries of current as a function of time at -80, -100 and -120 mV were well fitted by a single exponential function with recovery time constants from inactivation (τ_rec_). For example, τ_rec _for Ca_v_2.3 and Ca_v_2.3 + SmCa_v_β from -100 mV were, respectively, 71 and 168 ms (p < 0.05, Student t-test) (Figure 1E; see Table 1 for recovery time constants for the three potentials).

The mammalian (rat) β_2a _subunit (GB Acc. # M80545) increased current amplitude to a similar extent as the schistosome Ca_v_β subunit, shifted the steady-state inactivation curve in the hyperpolarizing direction, although to a lesser degree than the schistosome Ca_v_β subunit, slowed macroscopic inactivation, and shifted the I–V peak in the hyperpolarizing direction (Figure 1).

### Ca_v_2.3 currents run-down in the presence of SmCa_v_β in a manner independent of Ca^2+ ^but dependent on chelated forms of ATP and free Na^+^

**Figure 2:**
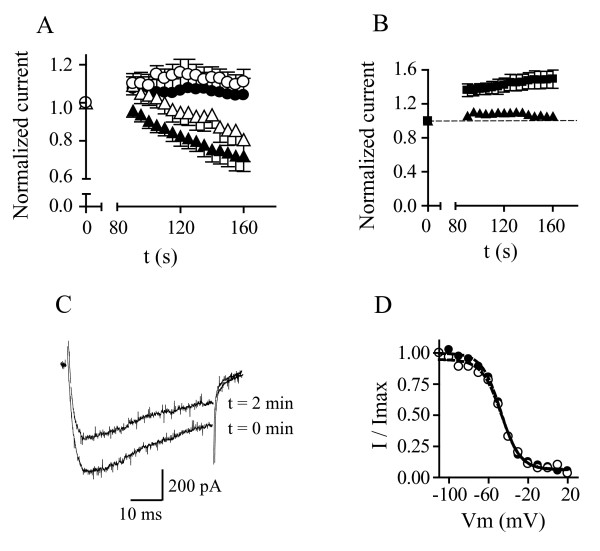
**SmCa_v_β-dependent run-down of Ca_v_2.3 currents**. *A*. Relative peak amplitude of currents produced by Ca_v_2.3 channels alone (circles) and in combination with SmCa_v_β (triangles), plotted as a function of time. Currents were normalized with respect to those measured immediately after rupturing the patch membrane (t = 0). Ca^2+ ^currents (filled symbols) and Ba^2+ ^currents (open symbols) are shown. Data points represent mean ± s.e.m. *B*. Relative amplitude of currents produced by Ca_v_2.3 in combination with Ca_v_β_2a _(squares) or SmCa_v_β_var _(triangles), plotted as a function of time. As in A, currents were normalized with respect of those at time 0, measured immediately after break-in. Data points represent mean ± s.e.m, N = 3 – 5. *C*. Ca^2+ ^currents generated by Ca_v_2.3 in the presence of SmCa_v_β at t = 0 min and at t = 2 min. Note that the kinetics of macroscopic inactivation remains unchanged. *D*. Voltage-dependence of inactivation (steady-state inactivation) at t = 0 min (filled circles) and at t = 2 min (open circles). Solid lines represent fits to the Boltzmann function.

Currents produced by Ca_v_2.3 progressively decrease in amplitude to approximately 75% of their initial values within ~2.5 minutes of establishing the whole-cell configuration, but only when SmCa_v_β is co-expressed (Figure 2A). Over the same time frame, no run-down was observed for currents produced by Ca_v_2.3 expressed alone, nor in the presence of the mammalian Ca_v_β_2a _subunit nor with the structurally atypical schistosome SmCa_v_β_var _subunit (Figure 2B). Substitution of CaCl_2 _by an equimolar concentration of BaCl_2 _did not prevent run-down, indicating that this phenomenon is Ca^2+ ^independent (Figure 2A).

We hypothesized that this rapid run-down could be caused by physical dissociation between SmCa_v_β and Ca_v_2.3. However, as the kinetics of inactivation and the steady state inactivation properties were identical before and after run-down (Figure 2C,D), it seems likely that the association between Ca_v_2.3 and SmCa_v_β remained intact in these conditions.

**Figure 3:**
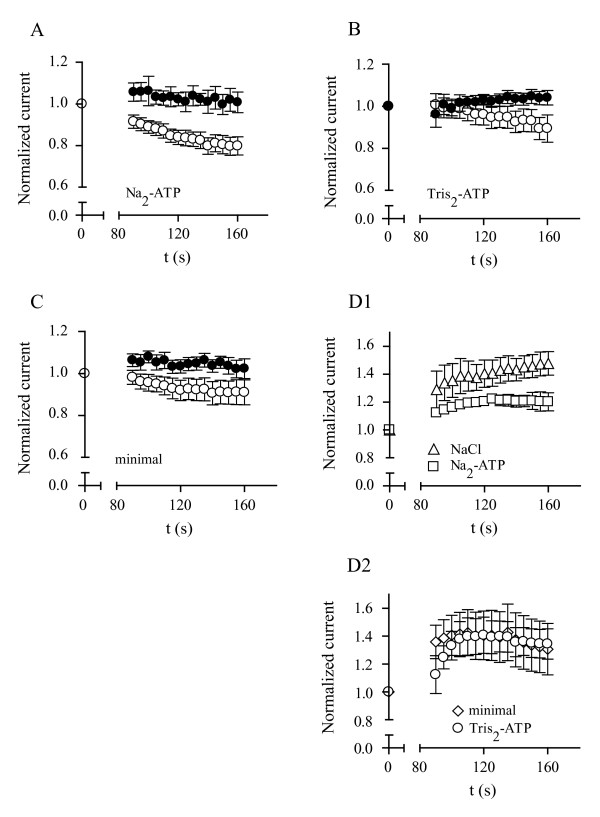
**Run-down of Ca_v_2.3/SmCa_v_β currents in the presence of intracellular forms of ATP**. Relative current amplitude of currents produced by Ca_v_2.3 (filled circles) channels alone and in combination with SmCa_v_β (open circles) plotted as a function of time in the presence of 5 mM Na_2_^+^-ATP in the patch-pipette solution (A), 5 mM Tris_2_-ATP (B), and using a "minimal" pipette solution, with no ATP (C). Currents were normalized with respect to those measured immediately after rupturing the patch membrane (t = 0). Panels D1 and D2 show current amplitude produced by Ca_v_2.3/SmCa_v_β_2a _channels as a function of time for all conditions explained above. Symbols are as shown on the figure. Values are means ± s.e.m, N = 3 – 8.

Since Mg^2+ ^is known to block Ca^2+ ^channels from the intracellular side, we replaced Mg^2+^-ATP with an equimolar concentration (5 mM) of Na^+^_2_-ATP, yet under these conditions we still observed significant run-down that, again, was dependent upon co-expression of the schistosome Ca_v_β subunit (Figure 3A). No run-down was observed in whole-cell patch-clamp experiments using a "minimal" internal solution without ATP (Figure 3C). Further experiments indicate that non-chelated forms of ATP (Tris_2_-ATP) do not mediate SmCa_v_β-dependent Ca_v_2.3 run-down (Figure 3B). Currents produced by Ca_v_2.3/Ca_v_β_2a _did not run-down in the presence of 5 mM intracellular NaCl, Na_2_-ATP, Tris_2_·ATP or in minimal intracellular solution (Figures 3D1 and 3D2).

**Figure 4:**
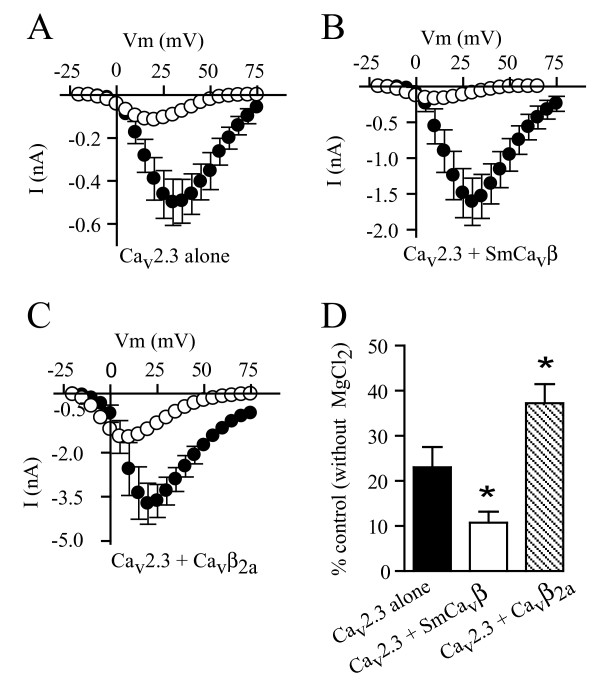
**Mg^2+ ^block of Ca_v_2.3 channels expressed alone and co-expressed with SmCa_v_β or Ca_v_β_2a_**. Current-voltage relationships from HEK cells expressing Ca_v_2.3 subunits alone (A), co-expressing Ca_v_2.3 and SmCa_v_β subunits (B) or co-expressing Ca_v_2.3 and Ca_v_β_2a _subunits (C) in minimal intracellular solution (filled circles) and in intracellular solution containing 5 mM MgCl_2 _(open circles). Peak currents generated by voltage steps from -20 mV to +75 mV in 5 mV steps from a holding potential of -80 mV are plotted. Data points represent mean ± s.e.m. *D*. Peak current amplitude in the presence of 5 mM internal MgCl_2 _relative to peak current amplitude in the absence of MgCl_2 _for Ca_v_2.3 subunits alone, co-expressed with SmCa_v_β subunits or co-expressed with Ca_v_β_2a _subunits. Asterisks denote statistically significant difference with respect to Mg^2+ ^block of Ca_v_2.3 channels alone (p < 0.05), N = 5 – 7.

**Figure 5:**
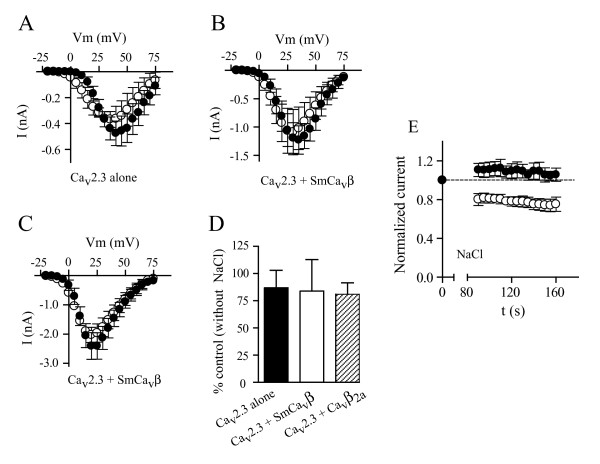
**Intracellular free Na^+ ^does not block Ca_v_2.3 channels expressed alone or co-expressed with SmCa_v_β or Ca_v_β_2a _but induces SmCa_v_β-dependent run-down of Ca_v_2.3 currents**. Current-voltage relationships from HEK cells expressing Ca_v_2.3 subunits alone (A), co-expressing Ca_v_2.3 and SmCa_v_β subunits (B) or co-expressing Ca_v_2.3 and Ca_v_β_2a _subunits (C) in minimal intracellular solution (filled circles) and in intracellular solution containing 5 mM NaCl (open circles). Peak currents generated by voltage steps from -20 mV to +75 mV in 5 mV steps from a holding potential of -80 mV are plotted. Data points represent mean ± s.e.m. (D) Peak current amplitude in the presence of 5 mM internal NaCl relative to peak current amplitude in the absence of NaCl for Ca_v_2.3 subunits alone, co-expressed with SmCa_v_β subunits or co-expressed with Ca_v_β_2a _subunits. (E) Relative current amplitude of currents produced by Ca_v_2.3 (filled circles) channels alone and in combination with SmCa_v_β (open circles) plotted as a function of time in the presence of 5 mM NaCl in the patch-pipette solution. Values are means ± s.e.m, N = 3 – 9.

Next we explored the possibility that this particular form of run-down was caused by the cations associated with the ATP molecule (Mg^2+ ^and Na^+^). Free Mg^2+ ^reduced currents produced by Ca_v_2.3 alone by about 75%, and by about 90% when SmCa_v_β was co-expressed (Figure 4); therefore, since it would be very challenging to distinguish Ca^2+ ^current decrease due to run-down from Ca^2+ ^current decrease due to blockade by internal Mg^2+^, we did not pursue run-down studies in the presence of free Mg^2+^. However, in contrast with Mg^2+^, free Na^+ ^did not suppress the activity of Ca_v_2.3 channels expressed alone or co-expressed with the schistosome SmCa_v_β or with the mammalian Ca_v_β_2a _subunit (Figure 5A–D). However, substituting Mg^2+^-ATP with NaCl resulted in significant run-down (Figure 5E), indicating Na^+^-dependent modulation of channels containing SmCa_v_β.

### The structural basis for run-down mediated by SmCa_v_β resides in its acidic N-terminal domain

**Figure 6:**
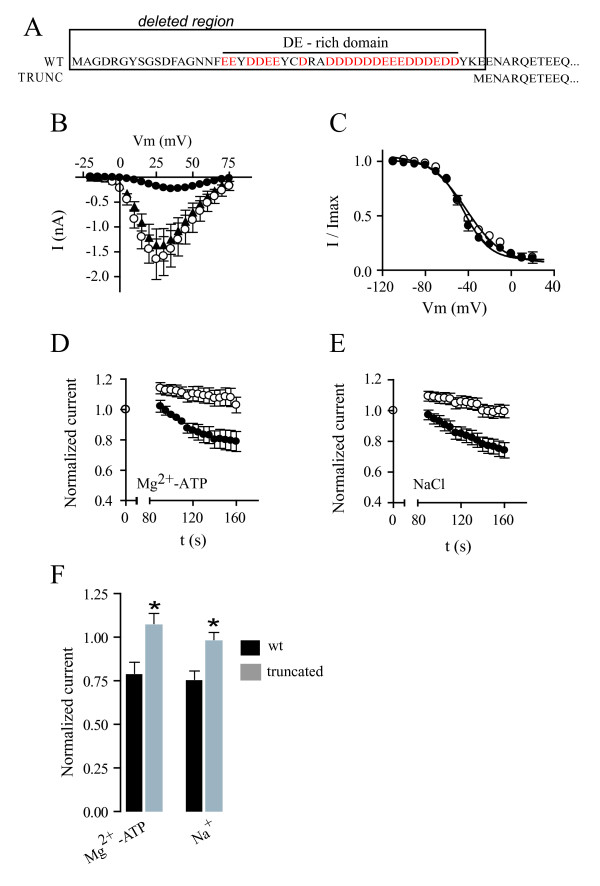
**The N-terminal domain of SmCa_v_β is the molecular substrate for Na^+ ^and Mg^2+^-ATP mediated block of Ca_v_2.3 currents**. *A*. Diagram showing the portion of the N-terminal domain, including a domain rich in aspartic acid and glutamic acid (DE-rich domain), that is removed by the truncation. Acidic residues are in red. *B*. Current-voltage relationships from HEK cells expressing Ca_v_2.3 alone (filled circles) and in combination with SmCa_v_β (open circles) or N-terminally truncated SmCa_v_β (filled triangles). Peak currents generated by voltage steps from -20 mV to +75 mV in 5 mV steps from a holding potential of -80 mV were measured. Data points represent mean ± s.e.m. N = 6–7. *C*. Voltage-dependence of inactivation (steady-state inactivation) for Ca_v_2.3 alone co-expressed with SmCa_v_β (filled circles) or with its N-terminally truncated version (open circles). Lines represent fits to the Boltzmann function. *D*. Relative peak current amplitude of currents produced by Ca_v_2.3 channels in the presence of internal 5 mM Mg^2+^-ATP co-expressed with the wild type SmCa_v_β subunit (filled circles) or with the N-terminally truncated SmCa_v_β subunit (open circles) plotted as a function of time. Currents were normalized with respect to those measured immediately after rupturing the patch membrane (t = 0). Data points represent mean ± s.e.m. N = 5. *E*. Relative current amplitude of currents produced by Ca_v_2.3 channels in the presence of internal 5 mM NaCl co-expressed with the wild type SmCa_v_β subunit (filled circles) or with the N-terminally truncated SmCa_v_β subunit (open circles) plotted as a function of time. Currents were normalized with respect to those measured immediately after rupturing the patch membrane (t = 0). Data points represent mean ± s.e.m. N = 10–13. *F*. Comparison between the relative current amplitudes produced by Ca_v_2.3 channels co-expressed with SmCa_v_β (in black) or with its N-terminally truncated version (in grey), in the presence of intracellular 5 mM Mg^2+^-ATP (left), or in the presence of 5 mM NaCl (right), after 2.5 minutes of establishing the whole-cell configuration. Asterisks denote statistical difference with respect to current amplitude for Ca_v_2.3 co-expressed with wild type SmCa_v_β (Student t test, p < 0.05).

The N-terminus of the SmCa_v_β subunit contains an atypical domain that is rich in glutamic acid and aspartic acid residues (Figure 6A). Since BLAST screens for similar domains in other proteins did not yield any hits, we set to investigate whether this N-terminal domain represents the structural base for the atypical modulation of the SmCa_v_β subunit. Furthermore, there is precedence for important modulatory effects on Ca_v _channels localizing to the N-terminal regions of β subunits [23-26]. To this end, we created a truncated version of SmCa_v_β that lacks the first forty-six amino acids containing this acid-rich domain (Figure 6A), and tested whether it still caused Ca_v_2.3 currents to run-down. The N-terminally truncated SmCa_v_β subunit enhanced Ca_v_2.3 currents, slowed their macroscopic inactivation and shifted their steady-state inactivation to the same extent as the wild type SmCa_v_β subunit (Figure 6B,C). However, unlike the wild-type version of SmCa_v_β, the N-terminally truncated SmCa_v_β subunit did not induce run-down of Ca^2+ ^currents within 2–3 minutes of establishing the whole-cell configuration with an internal solution containing either 5 mM Mg^2+^-ATP (Figure 6D,F), or 5 mM NaCl (Figure 6E,F).

## Discussion

In this study we set out to characterize the modulatory phenotype of a Ca_v_β subunit from the parasitic trematode *S. mansoni*. This β subunit is phylogenetically close to other Ca_v_β subunits [14]. The mammalian HEK-293 cell line was used as the expression system and the mammalian Ca_v_2.3 channel splice variant "d" as our "test" α_1 _subunit. This splice variant is susceptible to modulation by Ca_v_β subunits [27] and is the longest of all known versions of the Ca_v_2.3 subunit [28]. Our data show that SmCa_v_β exhibits similar modulatory phenotypes in HEK cells and *Xenopus *oocytes. Additionally, SmCa_v_β slows recovery from inactivation of Ca_v_2.3 currents using Ba^2+ ^as the charge carrier, i.e. in the absence of intracellular Ca^2+ ^accumulation. Using strontium as the charge carrier, mammalian and jellyfish Ca_v_β subunits also slow recovery from inactivation of this same channel in conditions in which Ca^2+ ^does not accumulate in the intracellular compartment [11]. However, it is important to note that recovery from inactivation appears to depend heavily on the identity of the Ca_v_α_1 _subunit being assayed: all jellyfish and mammalian Ca_v_β subunits delayed recovery from inactivation of Ca_v_2.3 channels, but accelerated the recovery of the jellyfish L-type Ca_v _channel [11].

### SmCa_v_β-dependent run-down of Ca_v_2.3 currents

While characterizing the modulatory phenotype of SmCa_v_β, we consistently observed a rapid run-down of Ca_v_2.3 currents only in the presence of SmCa_v_β. This contrasts with previous studies, in which run-down appears to occur in the α_1 _subunit, independently of accessory subunits [29-31]. Down-regulation of Ca^2+ ^currents caused by interaction of Ca_v_β subunits with intracellular proteins is well documented. For example, interaction of Ca_v_β subunits with small GTPases of the RGK family [32], with large GTPases of the dynamin family [33], or with the nuclear protein HP1 [34] results in down-regulation of Ca^2+ ^currents. Our data represent the first case, to our knowledge, of down-regulation of Ca^2+ ^currents by interactions between Ca_v_β subunits and chelated forms of ATP or Na^+ ^ions, which are likely relevant to platyhelminth physiology. Unlike previous studies, in which hydrolysable forms of ATP suppress Ca^2+ ^current run-down [35,36], here we show that two physiologically relevant, hydrolysable forms of ATP induce SmCa_v_β-mediated decrease in Ca_v_2.3 activity. The possibility that run-down was caused by Mg^2+ ^ions that dissociate from the ATP molecule was considered, but the fact that free Mg^2+ ^dramatically suppressed Ca_v_2.3 currents posed a significant challenge to explore this possibility. These results are reminiscent of the effects of Mg^2+ ^ions at a similar concentration on L-type Ca_v _channels, which occurs by their binding to a low-affinity site at the pore of the α 1 subunit [37]. Since ATP associates with Na^+ ^ions under physiological conditions [38], we also measured run-down of Ca_v_2.3/SmCa_v_β channels in the presence of intracellular Na^+^_2_-ATP. Because we detected significant run-down under these conditions, and knowing that the binding constant between Na^+ ^ions and ATP is relatively low, about 13 M^-1 ^(in contrast to 9000 M^-1 ^for Mg^2+^, [39]), we hypothesized that Na^+ ^ions that had dissociated from ATP were causing this run-down. Experiments using NaCl instead of Na^+^_2_-ATP confirmed this hypothesis.

Our study is not the first to show Ca_v_β-dependent run-down of non-L type Ca^2+ ^current. The mammalian Ca_v_β subunit appears to enhance run-down of the non-L-type, Ca_v_2.1 α_1 _subunit [40]. However, it is important to note that in that study, run-down occurred under the two-electrode voltage clamp configuration on *Xenopus *oocytes, where the connection between cytoplasm and plasma membrane remains largely intact. Therefore the mechanism(s) of run-down employed by the mammalian Ca_v_β subunit is likely to be distinct from that used by the schistosome Ca_v_β subunit.

### Molecular substrate for SmCa_v_β-dependent Mg^2+^-ATP and Na^+ ^sensitivity

Since the conventional SmCa_v_β subunit contains a string of acidic residues in the N-terminal domain that is not found in its mammalian counterparts, we generated a version of this subunit that lacked this region, to test whether this unique domain is the molecular substrate for this particular form of run-down. Deletion of this acidic, N-terminal fragment did not change the modulatory phenotype of SmCa_v_β on current amplitude, inactivation kinetics or steady-state inactivation. However, in contrast to our results with wild type SmCa_v_β, Ca_v_2.3 currents did not run down when co-expressed with this mutated subunit, using Ca^2+ ^as the charge carrier and in the presence of intracellular free Na^+ ^or Mg^2+^-ATP. It seems likely that sensitivity to intracellular Mg^2+^-ATP and free Na^+ ^resides in all or part of the string of acidic residues of the SmCa_v_β subunit.

### Physiological relevance

Several reports have shown that the Ca^2+ ^currents of platyhelminth muscle and nerve cells are very labile, running down within minutes or even seconds after establishing the whole-cell patch-clamp configuration. In previous work, the intracellular solution used to record voltage-gated Ca^2+ ^currents from muscle cells of *S. mansoni *was titrated with NaOH to bring the pH to a physiological value [41]. We have empirically calculated that this action would bring the concentration of Na^+ ^to approximately 19 mM, which is sufficient to reduce Ca^2+ ^currents modulated by SmCa_v_β significantly, according to our data. Similarly, Ca^2+ ^currents recorded from isolated muscle cells of the free-living flatworm *Bdelloura candida *run-down within 20 seconds of establishing the whole-cell configuration [42]: It is tempting to speculate that this run-down was caused by the relatively high concentration of NaCl (30 mM) added to the intracellular solution in these experiments.

Our previous studies have identified a different schistosome Ca_v_β subunit (SmCa_v_β_var_; [14]), which does not exhibit the hallmark action of Ca_v_β subunits, namely, to increase Ca^2+ ^current density. Together with the data presented here, this raises the question of whether schistosomes employ unique strategies to modulate excitability via atypical modulation of HVA Ca_v _channels, information that could be useful in the design of targeted therapies to treat schistosomiasis.

## Conclusion

We have identified novel functions for a schistosome Ca_v_β subunit, namely to confer Ca^2+ ^currents with sensitivity to intracellular Mg^2+^-ATP and Na^+ ^ions, which translates into a reduced ability on the part of this Ca_v_β subunit to increase Ca^2+ ^currents. We conclude that the molecular basis for this atypical sensitivity to both Mg^2+^-ATP and Na^+ ^ions resides in a domain or domains located within the first forty-six amino acids of SmCa_v_β, which contains a string of twenty-two aspartic and glutamic acid residues not present in other Ca_v_β subunits.

## Methods

### Materials

Tissue culture dishes were purchased from Corning (NY, USA), Dulbecco's modified Eagle's media (DMEM) was purchased from Invitrogen, poly-L-lysine and ATP salts were purchased from Sigma. The transfection reagent, Tfx, was purchased from Promega. Restriction enzymes were from NEB, and oligonucleotide primers were from MWG Biotech.

### Preparation of eukaryotic expression plasmids encoding SmCa_v_β

Using standard methods, we cloned all Ca_v_β subunits into the pXOOM vector [43], which is optimized for expression of inserts in mammalian cells (under control of a cytomegalovirus promoter), and contains the gene for green fluorescent protein (GFP) as a marker for transfection. For SmCa_v_β, the insert from the original SmCa_v_β clone in pCR4-TOPO (Invitrogen) was amplified using Phusion high-fidelity DNA polymerase (NEB). Primers were designed against the beginning and end of the coding regions of the sequence, and included appropriate restriction sites for insertion into pXOOM. The primers were: Forward: 5'-GGAAGCTTATGGCTGGTGATCGAGGATATTCA-3', which includes two G residues and a *Hind III *site at the 5' end; and Reverse: 5'-GGGCGGCCGCTTAAATCATGATTGAACCTTGACGA-3', which includes two G residues and a *Not I *site at the 5' end. Following an initial 98° denaturation for 30 seconds and 25 cycles of 98° for 10 s, 68° for 30 s, and 72° for 2 min, the reaction was purified over a QiaQuick spin column (Qiagen), and digested with *Hind III *and *Not I*. The digested band was gel-purified using Quantum Prep Freeze-n-Squeeze columns (BioRad), Pellet Paint (Novagen) was added as carrier, and the product was ethanol-precipitated and ligated to pXOOM that had been digested with *Hind III *and *Not I *and gel-purified. All constructs were sequenced to verify the absence of PCR errors.

### Cell culture and transfection of HEK293-Ca_v_2.3 cells

HEK293 cells stably transfected with Ca_v_2.3d [44] were cultured in DMEM supplemented with L-glutamine, glucose and 10% foetal bovine serum in a humidified atmosphere (95%) at 5% CO_2 _and 37°C. Cells were used for up to 20 passages and were split every 2–4 days. For electrophysiological recordings, cells were seeded in Petri dishes coated with poly-l-lysine, and transfection of auxiliary β subunits was performed with Tfx on cells at a confluence of 50–60%, using 1 μg of the construct and a DNA: Tfx ratio of 1:2. Cells exhibiting green fluorescence were used for further study.

### Construction of a N-terminal deletion mutant of the SmCa_v_β subunit

To generate a mutant subunit lacking amino acids 2–46 of the N terminus domain, a diluted sample of the SmCa_v_β clone was used as template for amplification of the truncated insert by PCR. The forward primer (5'-GGGGATCCATGGAAAATGCTCGTCAGGGAACGG-3') was designed to bind to the SmCa_v_β clone, starting from nucleotide 142. This forward primer contains a start codon, a Kozak sequence and a BamHI restriction site. The reverse primer was the same one used to amplify the full-length sequence. These PCR products were inserted into pXOOM and transformed into *E. coli*. Clones were sequenced to verify the deletion and to detect possible PCR errors.

### Electrophysiology

Whole-cell recordings were obtained at room temperature 24 hours following transfection using an Axopatch 200B (Molecular Devices). Cell capacitance was 12–25 pF. Series resistance was compensated by 70%. Voltage pulses from -20 mV to +70 mV were delivered in 5 mV increments from a holding potential of -80 mV. Data were acquired at sampling intervals of 50 μs and filtered at 5 kHz during acquisition. The pipette solution contained (mM): cesium methane sulfonate (110), HEPES (10), EGTA (9), Mg^2+^-ATP (5); pH (CsOH) 7.3, with variations, as noted. The bath solution contained (mM): CaCl_2 _(10), TEA-Cl (160), HEPES (10), EGTA (0.1); pH (TEA-OH) 7.4. Patch pipettes were pulled from borosilicate glass and fire polished before each experiment. To ensure a fast dialysis of the intracellular compartment, only pipettes with resistances between 0.8 and 1.2 MΩ were used. Membrane seals were obtained by applying negative pressure. All experiments were performed at room temperature (22°C). The voltage-dependence of steady state inactivation was determined by measuring the peak current evoked with a depolarising pulse to elicit the maximum current as a function of the voltage of a preceding 1.5 s pre-pulse test (between -110 and +20 mV). Steady-state inactivation curves were fitted by a sigmoid (Boltzmann) distribution of the form:

F (V) = I_max_/{1 + exp [V_0.5 _- V)/K]}

Where *I*_*max *_is the maximal current, *V *is the pre-pulse voltage, *K *is the slope factor and *V*_0.5 _is the voltage at which inactivation is half-maximal.

To study inactivation as a function of time, the decaying phases of the inward currents evoked by a test pulse to +20 or +30 mV were fitted to a double exponential equation of the form: I (t) = I_0 _+ I_1 _exp (-t/τ_1_) + I_2 _exp (-t/τ_2_), where τ_1 _and τ_2 _represent the fast and the slow time constants of inactivation, respectively, *I*_1 _and *I*_2 _represent the relative contribution of each component to inactivation and *I*_0 _is the offset. To assess the rate of recovery from inactivation from the closed state, a two-pulse protocol was used. Two test pulses to +20 or +30 mV were separated by a recovery step to -80, -100 or -120 mV for varying amounts of time (from 10 ms to 1 second). The length and voltage of the first test pulse was adjusted accordingly for each particular channel combination. For example, currents produced by Ca_v_2.3 channels alone were maximal at approximately +30 mV and inactivated completely within 100 ms, whereas currents produced by Ca_v_2.3/SmCa_v_β were maximal at approximately +20 mV and required several seconds to inactivate fully. In the latter case, recovery from inactivation was measured using Ba^2+ ^instead of Ca^2+ ^as the charge carrier (Ba^2+ ^currents inactivate faster than Ca^2+ ^currents produced by Ca_v_2.3 [45]), thereby decreasing the need to use an excessively long depolarising first pulse protocol, which could compromise cell viability. The currents evoked by the second pulse of this double-pulse protocol were normalized to the currents produced by the first pulse and plotted against the duration of the inter-pulse interval.

### Statistical analyses

Statistical comparisons were carried out using the Student t-test. Data are presented as means ± s.e.m. Number of repeats is indicated in parentheses.

## Authors' contributions

VSR carried out the molecular and electrophysiological studies, made substantial contributions to conception and experimental design, and drafted the manuscript. TS provided the HEK cell line stably transfected with Ca_v_2.3 and made a significant contribution to the interpretation of the data. RMG carried out molecular work and contributed to experimental design as well as to the writing of the manuscript. All authors read and approved the final manuscript.
